# Association of a healthy ageing index with health-related outcomes in a multi-ethnic cohort from Singapore

**DOI:** 10.1186/s12877-024-05099-7

**Published:** 2024-06-11

**Authors:** Nazira Binte Muhammad Fauzi, Xiangyuan Huang, Ling Jie Cheng, Nan Luo, Saima Hilal

**Affiliations:** 1 Saw Swee Hock School of Public Health, National University of Singapore and National University Health System, Tahir Foundation Building, 12 Science Drive 2, #10-03U, 117549 Singapore, Singapore; 2 Department of Pharmacology, National University of Singapore, Singapore, Singapore

**Keywords:** Ageing, Population-health, Chronic diseases, Multiethnic, Asian

## Abstract

**Background:**

The global population is ageing rapidly and it is important to promote healthy ageing. The Healthy Ageing Index (HAI) is a comprehensive measure of health, but there is limited research on its association with other age-related outcomes. The management of an aging population necessitates considerations even among generally healthy adults, as age-related diseases often remain unaccounted for until later stages of life. This study explores the association of risk factors with HAI and its association with peripheral artery disease (PAD), muscle strength, health-related quality of life (HRQoL), and psychological distress in the Singapore Multi-Ethnic Cohort study.

**Methods:**

This cross-sectional study involved 1909 participants (median (Q1, Q3) age: 53 (48, 60) years and 59.3% females) from Singapore Multi-Ethnic Cohort study. The risk factors of HAI included age, gender, ethnicity, education level, smoking, alcohol consumption, employment, BMI and past medical histories. PAD was assessed using ankle-brachial index (ABI), handgrip strength (HGS), HRQoL with the EQ-5D-5 L questionnaire and psychological distress via the Kessler Psychological Distress Scale (K10). HAI components were assessed using relevant marker tests.

**Results:**

Older age, Malay and Indian ethnicities, unemployment, high BMI and histories of CHD, hypercholesterolaemia, tumours and TIA/stroke were associated with lower HAI scores indicative of poorer health. Higher HAI scores were associated with females and higher education levels. Lower HAI scores were significantly associated with low ABI, high K10 scores, mobility and anxiety/depression dimensions of EQ-5D-5 L.

**Conclusion:**

The most important factors associated with HAI were age, sex, ethnicity, education, unemployment, BMI and a history of health conditions. Lower HAI scores were significantly associated with PAD, lower HRQoL and psychological distress. Thus, the HAI demonstrates promise as an evaluation method for assessing PAD, overall muscle strength and HRQoL in a population-based setting.

**Supplementary Information:**

The online version contains supplementary material available at 10.1186/s12877-024-05099-7.

## Introduction

Ageing is a biological process influenced by various factors that affect the health and lifestyle of an individual. It is occurring more rapidly now than ever, with some countries such as Japan already in the advanced stages of changing population demographics [1]. This poses a problem, as countries must be prepared to tackle the health and social issues of an increasingly ageing population [2]. According to the World Population Prospects 2019, by 2050, 1 in 6 people will be older than 65 [3]. Singapore has a rapidly ageing population whereby by 2030, an estimated 1 in 4 Singaporeans will be 65 and older [4]. There is a great need to focus on promoting healthy ageing and the prevention of age-related diseases to prepare for the increase in older adults in the population [5].

As individuals progress in age, they become increasingly susceptible to a myriad of comorbidities, cognitive deterioration, and physical decline. This susceptibility contributes to the onset of age-related conditions, encompassing hypertension, frailty, metabolic dysregulation, and cognitive impairment. Declining muscle strength correlates with diminished levels of autonomy, exerting a notable impact on the overall well-being and quality of life among the elderly [6–9]. Cognitive dysfunction and frailty, traditionally associated with advanced age demographics, manifest even among individuals as young as 50 years old [10]. Shifts in dietary habits and lifestyle patterns notably augment the incidence and progression of age-associated illnesses among younger individuals, thereby necessitating tailored therapeutic approaches for effective management [11]. Therefore, it is crucial to identify comprehensive evaluation methods that capture multiple dimensions of health and functioning, which can lead to understanding the association between healthy ageing and age-related diseases.

However, there is a lack of consensus on the definition of healthy ageing, and previous studies have developed various indices with different components to evaluate healthy ageing [12–15]. One such index is the Healthy Ageing Index (HAI), a summary measurement based solely on physiological parameters from five physiological systems – cardiovascular, respiratory, metabolic, urinary and neurological systems [16, 17]. The development of the HAI was prompted, in part, by the limitations of existing comorbidity indices such as the Charlson comorbidity index in effectively capturing the medical needs of relatively well-functioning older adults who do not exhibit clinically recognisable diseases. Consequently, Newman et al. first introduced the “Physiologic Index of Comorbidity” (PIC), which proved capable of detecting subclinical diseases in older adults [16].

The HAI is a modified form of PIC that incorporates more easily accessible tests in community settings. For instance, systolic blood pressure is used as a surrogate for carotid intima-media thickness, while the digit symbol substitution test or the Modified Mini-Mental Status Examination (MMSE) are used to assess brain white matter [17]. Studies have shown that HAI serves as a reliable indicator for predicting mortality, mobility issues and incident cardiovascular disease [17–20]. The HAI score has been found to increase with age, indicating its usefulness as a summary marker for ageing [20].

Despite the global trend of aging populations, there remains a scarcity of research exploring the association of the Healthy Ageing Index (HAI) with age-related outcomes, particularly in relatively healthy adult populations. This represents a notable gap in the existing evidence, as managing the health and well-being of middle-aged adults may be critical for promoting healthy aging trajectories and preventing the onset of age-related diseases in later years.

Understanding the relationship between HAI and health outcomes can provide valuable insights into the impact of ageing on different aspects of health and inform interventions and strategies to promote healthy ageing and improve quality of life in older adults. Furthermore, the HAI has the potential to serve as a non-invasive screening method, enhancing accessibility and feasibility in identifying individuals at risk for age-related diseases. This aspect is particularly significant as younger adults are increasingly affected by such conditions today [21]. By utilising the HAI, earlier identification of these risks can be achieved, allowing for timely intervention and support.

With populations aging and demographics shifting, countries like Singapore are increasingly prioritizing active and successful aging strategies to meet the needs of their aging citizens [22]. The concept of successful aging, as proposed by Rowe and Kahn [23], emphasizes the importance of physical, psychological, and social engagement in life. There is, thus, a lack of robust evidence regarding the HAI in the general adult population from Singapore, particularly among individuals younger than 65. According to the National Population Health Survey conducted in 2020 [24], chronic diseases such as hypertension and poor mental health exhibit a higher prevalence among younger adult age groups aged 30–39 and 40–49 compared to previous years.

Peripheral artery disease (PAD) is prevalent among adults aged 60 and above, yet early detection is infrequent [25]. PAD thus, offers opportunities for early detection and insights into age-related diseases. The intricate interconnections between age-related diseases like PAD, overall muscle strength, and their impact on quality of life and psychological distress provide a comprehensive measure of aging and its effects on overall well-being. This study aims to investigate the association of risk factors with HAI and how the multidomain HAI is associated with outcomes that align with this holistic approach to successful aging, focusing on factors affecting physical, psychological, and social aspects of life. The outcomes selected for this study, namely PAD, muscle strength, health-related quality of life (HRQoL), and psychological distress, are directly pertinent to these factors.

## Methods

### Data availability

The datasets used and/or analysed during the current study available from the corresponding author on reasonable request.

### Study population

This cross-sectional study utilised data from the Singapore Multi-Ethnic Cohort revisit, a population-based prospective study aimed at studying the determinants and risk factors of various chronic health conditions in three main ethnic groups; Chinese, Malay and Indian [26].

During the baseline phase (Phase 1) spanning from 2004 to 2010, the Multi-Ethnic Cohort recruited a total of 14,465 participants. These participants were adult Singapore citizens or long-term residents aged between 21 and 75 years, with no existing chronic diseases such as cancer, cardiovascular diseases, renal failures, and mental illnesses. Of the baseline participants, 28% were not contactable (i.e. contact details changed and no updated information available, or frequently travelling, or access to household denied and unable to contact despite six attempts at household visitation) and 2.5% were confirmed to have been lost to follow-up (i.e. deceased, migrated, declined follow-up, lost mental competence to give consent to continue the research, institutionalized or physically unfit to participate). Of the contactable participants, 60% (*N* = 6101) agreed to participate in the follow-up survey between 2011 and 2016. At both baseline and revisit, a comprehensive questionnaire collected data on demographics, socioeconomic status, medical and family history, as well as lifestyle factors including diet and smoking. Additionally, health screenings were performed at both time points, encompassing measurements such as anthropometric data, blood pressure, blood glucose, and blood lipids. Data for analysis was taken from the revisit (follow-up).

**Fig. 1:**
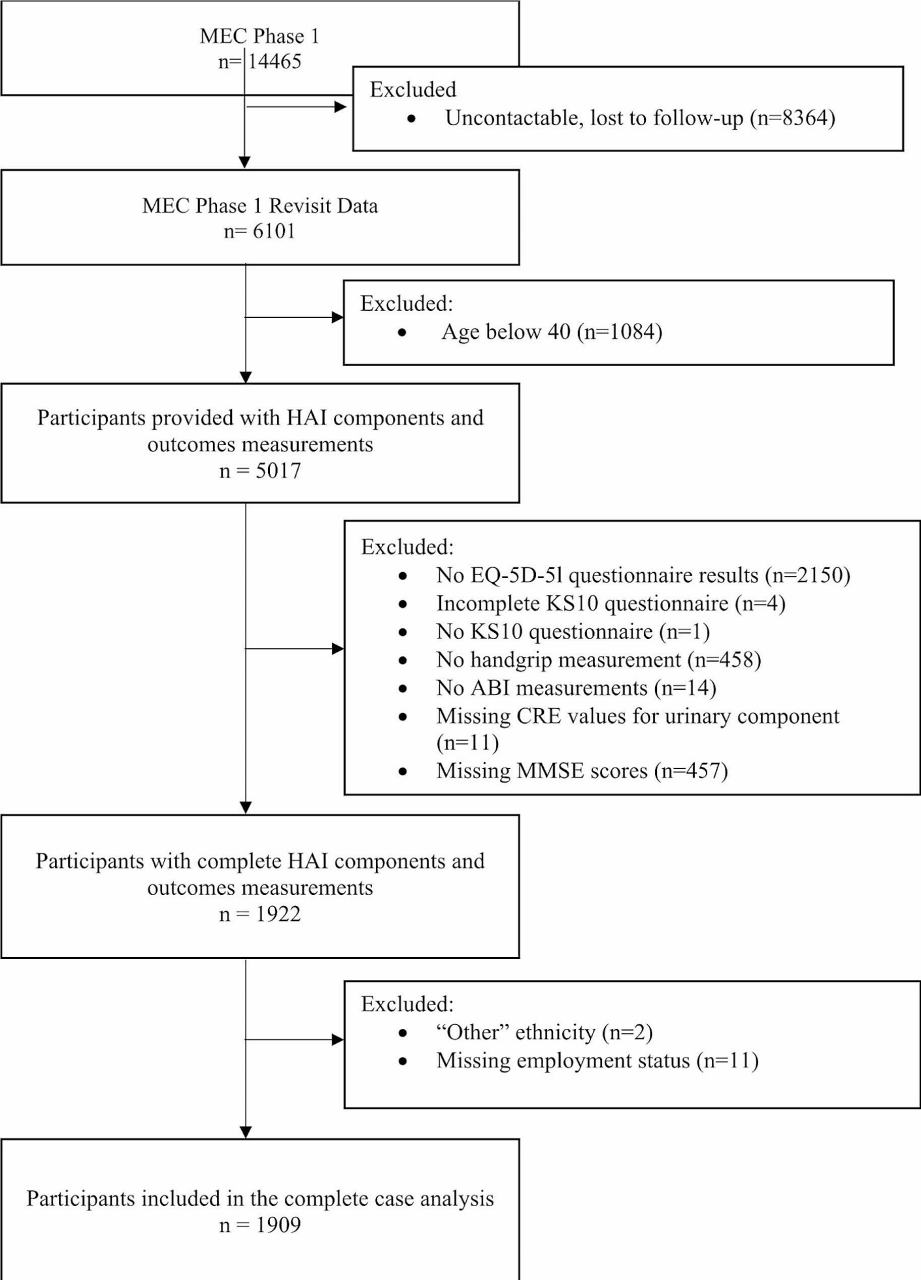
Flowchart of eligible MEC participants included in this study’s analyses

Of these participants, 1,084 individuals were excluded as they were below the age of 40, the designated threshold for early disease screening. Furthermore, 3,095 participants lacked complete information on HAI components and outcome measurements. Participants of ethnicities other than Chinese, Malay and Indian were also excluded due to the small sample size (*n* = 4). Furthermore, 11 individuals with missing sociodemographic information were excluded, resulting in a final analysis of 1909 participants (Fig. 1).

### Healthy ageing index

The HAI comprises data from markers of five physiologic systems: systolic blood pressure (cardiovascular), pulmonary problems (respiratory), fasting blood glucose (metabolic), creatinine levels (urinary) and MMSE score (neurological).

### Systolic blood pressure

Blood pressure was measured twice in the seated position by an automated digital monitor. The average of the two measurements was used to construct the HAI. Participants were classified into three groups according to clinical cut-off points: 0 = ≥ 143 mmHg, 1 = 126–143 mmHg and 2 = < 126 mmHg [18, 27, 28]. Participants who had a self-reported history of hypertension or were taking antihypertensive medication were assigned 0 points.

### Pulmonary problems

Self-reported pulmonary disease was employed as a proxy marker to assess the effects of chronic lung conditions [29, 30]. Individuals without a history of pulmonary disease were categorized into the healthiest group, receiving a score of 2. Participants who self-reported a history of chronic lung diseases, encompassing conditions such as asthma, emphysema, chronic bronchitis, chronic obstructive pulmonary disease, and pulmonary tuberculosis, were assigned 0 points.

### Fasting blood glucose

Serum fasting blood glucose levels were categorized into three groups according to clinical cut-off points: 0 = ≥ 7.0 mmol/L, 1 = 5.6-7.0 mmol/L, and 2 = ≤ 5.5 mmol/L [31]. Participants who had a self-reported history of diabetes or were undergoing diabetic medication treatment were assigned 0 points.

### Creatinine

Serum creatinine levels were employed as an indicator of urinary function and categorized into sex-specific groups. For men: 0 = ≥ 114.9 mmol/L, 1 = 97.2-114.9 mmol/L, and 2 = < 97.2 mmol/L. For women: 0 = ≥ 88.4 mmol/L, 1 = 70.7–88.4 mmol/L, and 2 = < 70.7 mmol/L.

### Mini-mental state exam

Cognitive function was assessed using the MMSE, a 30-point questionnaire commonly utilized to gauge cognitive impairment. Participants were categorized based on cut-off points previously established in Singaporean Chinese older adults [32, 33]: 0 = ≤ 25 (indicating cognitive impairment) and 2 = ≥ 26 (representing cognitive normalcy).

The HAI was formulated by summing the scores of each of the five components, resulting in a total score that spans from 0 (indicating the least healthy) to 10 (signifying optimal health). Data values for each component were grouped into three categories: the healthiest group received a score of 2, the intermediate group scored 1, and the least healthy group scored 0. The cutoff values utilized in this study were adopted from prior research for systolic blood pressure, creatinine levels, and MMSE scores [18, 27–33]. Clinical cutoff points were applied for fasting blood glucose (Supplementary Table 1).

### Risk factors

Sociodemographic data encompassing year of birth, gender, ethnicity (Chinese, Malay, Indian), and employment status were gathered through standardized questionnaire-based interviews at both baseline and the Revisit. Education level was categorized into three groups: primary level (primary education and below), secondary level, and tertiary level (post-secondary and above). Weight was measured using SECA digital scales (SECA700 series), while height was measured without shoes using a portable stadiometer (SECA200 series) [34]. Body mass index (BMI) was calculated as the ratio of body weight (in kg) to the square of height (in meters). BMI was classified according to the WHO Asian BMI classification: underweight (< 18.5 kg/m²), normal/low risk (≥ 18.5 - <23.0 kg/m²), moderate risk (≥ 23.0 - <27.5 kg/m²), and high risk (≥ 27.5 kg/m²) [35, 36].

Lifestyle factors were also assessed. Smoking status was categorized as ever-smoker (including current and past smokers) or non-smoker. Alcohol consumption status was classified as alcohol drinkers (including current and past drinkers) or non-drinkers. These details were collected during the revisit. Additionally, participants were queried about their past medical history during the revisit, including occurrences of coronary heart disease (encompassing heart failure, heart attack, history of angioplasty, insertion of balloons and stents, and valve prolapse), hypercholesterolemia, tumors of any type, and transient ischemic attack/stroke.

### Outcome measurements

Outcomes were treated as binary variable based on clinical cut-off points or selected from previous research (Supplementary Table 2).

### Ankle-brachial index (ABI)

Ankle-Brachial Index (ABI) measurements were conducted utilizing sphygmomanometers (Accoson, UK). The measurements were taken from the brachial artery in both arms and from the dorsalis pedis or posterior tibial artery in both ankles while the participants were in the supine position. For each location, systolic blood pressure measurements were obtained and repeated once for accuracy. The ABI was calculated for each leg individually, bilaterally, as the ratio of the systolic blood pressure in the ankle to the highest systolic blood pressure in the arm. The ABI was separately calculated for each leg [37]. To define peripheral artery disease, the lower ABI value between the two legs was considered. Peripheral artery disease was defined as an ABI of ≤ 0.9 [38].

### Handgrip strength (HGS)

HGS was measured using TAKEI A5401 Hand-held Electronic Dynamometer (Taheel Technology, Saudi Arabia). Participants were given instructions to stand upright with their arms naturally hanging down. They were then directed to hold the dynamometer and grip it without any arm swinging, with the measurement capturing their maximum handgrip strength [28]. HGS was measured three times for each hand and repeated, resulting in a total of six measurements. The average HGS was then calculated from these six measurements. HGS was considered as a binary variable for analysis. Low HGS was defined according to Asian Workgroup for Sarcopenia criteria [33, 39]: <28 kg for men and < 18 kg for women.

### EQ-5D questionnaire

The EQ-5D-5 L self-report questionnaire, developed by the Euro-QoL group addresses five dimensions: (i) mobility, (ii) self-care, (iii) usual activities, (iv) pain/discomfort and (v) anxiety/depression [40, 41]. These dimensions are evaluated through five response levels: (i) no problems, (ii) slight problems, (iii) moderate problems, (iv) severe problems, and (v) extreme problems. Both the English and Chinese versions of this questionnaire have been validated for use in Singapore [42–44]. The EQ-5D-5 L index values can be derived using preference weights from the general population, reflecting the severity of the associated health state. However, as the preference weights specific to the Singapore population were not available, the index score was calculated using a crosswalk mapping function to EQ-5D-3 L value sets that have been developed for Singapore, ranging between − 0.769 (indicating death) and 1 (representing full health), with higher scores indicating a higher level of utility [45, 46].

Each dimension was considered a binary variable, categorizing the presence of any problems as yes/no. The response levels ranging from slight problems to extreme problems were grouped into a single outcome described as “having problems”.

### Kessler psychological distress scale (K10)

The K10 questionnaire comprises ten questions designed to evaluate anxiety and depressive symptoms experienced in the most recent four weeks. Responses to these questions are assessed using a five-point scale: (i) None of the time, (ii) A little of the time, (iii) Some of the time, (iv) Most of the time, and (v) All of the time. Scores on the K10 questionnaire range from 10 to 50, with lower scores indicating better mental well-being. The summed scores can be classified into four categories based on previous research: (i) the individual is likely well: 10–19; (ii) mild psychological distress: 20–24; (iii) moderate psychological distress: 25–29; and iv) severe psychological distress: 30–50 [47, 48]. Subsequently, participants were categorized into two groups: those classified as well (with scores ≤ 19) and those experiencing mild-to-severe psychological distress (with scores ≥ 20).

### Statistical analysis

Descriptive statistics included counts and percentages for categorical variables (gender, ethnicity, education level, employment status, smoking status, alcohol consumption, history of coronary heart disease (CHD), hypercholesterolemia, tumor, stroke), and median along with the first and third quartiles for continuous variables (age, BMI, HAI, ABI, HGS, K10, and MMSE). The distribution of risk factors were also presented across three ethnicities and differences were presented using chi-square for categorical variables and Kruskal-Wallis test for continuous variables.

To explore the cross-sectional relationships between risk factors and HAI, linear regression models were employed, and HAI was treated as a continuous variable.

In Model I, univariate analysis was performed where each factor was individually included without adjustments for covariates. Model II incorporated all sociodemographic factors (age, gender, ethnicity, education level), lifestyle factors (employment status, BMI, smoking status, and alcohol consumption), as well as comorbidities (history of CHD, hypercholesterolemia, tumor, and stroke).

Binary logistic regression models were employed to investigate associations between HAI and the outcome measures. Model I encompassed univariate analysis without any covariate adjustments. Model II was adjusted for sociodemographic factors. Model III included variables from Model II with additional adjustments for lifestyle factors (employment status, BMI, smoking status, and alcohol consumption). Model IV comprised all variables from Model III with further adjustments for any history of comorbidities.

The models were adjusted for these variables (sociodemographic factors, lifestyle factors, history of comorbidities) based on prior research that showed their influence on HAI [18, 30, 32]. A p-value of < 0.05 was regarded as statistically significant. All statistical analyses were carried out using STATA version 17.

## Results

Table 1 provides an overview of the characteristics of the study population. The median (Q1, Q3) age of the study participants was 53 years (48, 60) with a maximum age of 86 years. Among the participants, 1119 (59.3%) were women. The ethnic composition of the study population consisted of 757 (39.7%) ethnic Chinese, 478 (25.0%) Malays and 674 (35.3%) Indians. The participants in the study had a high cognitive function, and a few had a diagnosis of peripheral arterial disease, with the median (Q1, Q3) MMSE scores and ABI being 29 (27, 30) and 1.07 (1.01, 1.12). The majority of participants reported low psychological distress based on K10 scores. Education beyond the primary level, employment, and a healthy BMI were prevalent among the study participants, although a history of hypercholesterolemia was common. The HAI scores of the participants ranged from 1 to 10, with a median (Q1, Q3) score of 8 (6,10), indicating a generally healthy study population.

**Table 1:** Baseline characteristics of included participants (*N* = 1909)

Characteristics	*n* (%) / Median (Q1, Q3)
Age (min age = 40)	53 (48, 60)
Female	1119 (59.3)
Ethnicity	
Chinese	757 (39.7)
Malay	478 (25.0)
Indian	674 (35.3)
Education	
Primary	547 (28.7)
Secondary	781 (40.9)
Tertiary	581 (30.4)
Full time/part-time employment	1290 (67.6)
Smoking	386 (20.2)
Alcohol consumption	362 (19.0)
BMI (kg/m^2^)	25.3 (22.5, 28.7)
History of CHD^a^	162 (8.5)
History of hypercholesterolaemia	814 (42.6)
History of tumour of any type	36 (1.9)
History of TIA/stroke	21 (1.1)
HAI score	8 (6,10)
ABI	1.07 (1.01, 1.12)
HGS (kg)	22.7 (17.9, 29.6)
K10 score	12 (10, 16)
MMSE score	29 (27, 30)

ABI: Ankle-brachial index, BMI: Body Mass Index, CHD: Coronary Heart Disease, HAI: Healthy Ageing Index, HGS: Handgrip strength, K10: Kessler10 Psychological Distress Scale, MMSE: Mini-Mental State Examination, TIA: Trans-ischemic attack.

^a^ CHD: Heart failure, heart attack, history of angioplasty, insertion of balloons and stents and valve prolapse.

Supplementary Table 3 displays the prevalence of sociodemographic factors, lifestyle factors, and comorbidities history across the ethnic groups. Compared to the Chinese ethnicity, both Malays and Indians exhibit a higher frequency of CHD and hypercholesterolemia, as well as higher BMI. In contrast, the Chinese ethnicity demonstrates a higher prevalence of alcohol consumption. However, variables such as HAI score, ABI, HGS, K10 score, and MMSE score do not exhibit significant variations among the ethnic groups. Table 2 shows the associations between risk factors and HAI scores based on linear regression models. Model I demonstrated several significant associations with low HAI scores. These associations encompassed older age, unemployment, BMI levels classified as moderate and high risk, history of CHD, having a history of transient ischemic attack/stroke and hypercholesterolemia, and being of Malay or Indian ethnicity compared to Chinese. Conversely, higher education levels, and alcohol consumption, were linked to higher HAI scores. After implementing multivariate adjustments in Model II, the significant associations persisted for older age, unemployment, higher education, moderate and high-risk BMI levels, history of hypercholesterolemia, history of CHD and TIA/stroke and being of Malay or Indian ethnicity compared to Chinese. Notably, after adjustment, it was observed that women had higher HAI scores as well as a history of tumours of any type emerged as significantly associated with lower HAI scores,

**Table 2:** Association of sociodemographic, lifestyle and history of comorbidities and HAI

	Model I		Model II	
	β (95% CI)	*p*	β (95% CI)	*p*
Age, y	-0.10 (-0.10, -0.09)	**< 0.001**	-0.07 (-0.08, -0.06)	**< 0.001**
Female	0.015 (-0.1630.19)	0.872	0.21 (0.04, 0.37)	**0.018**
Ethnicity				
Chinese	1.00 (ref)		1.00 (ref)	
Malay	-1.18 (-1.40, -0.97)	**< 0.001**	-0.50 (-0.70, -0.31)	**< 0.001**
Indian	-1.21 (-1.40, -1.02)	**< 0.001**	-0.56 (-0.73, -0.39)	**< 0.001**
Education				
Primary	1.00 (ref)		1.00 (ref)	
Secondary	1.20 (1.00, 1.40)	**< 0.001**	0.76 (0.59, 0.93)	**< 0.001**
Tertiary	1.81 (1.60, 2.02)	**< 0.001**	0.93 (0.73, 1.13)	**< 0.001**
Unemployed	-0.90 (-1.08, -0.71)	**< 0.001**	-0.29 (-0.46, -0.13)	**0.001**
Smoker	-0.012 (-0.29, 0.27)	0.932	-0.15 (-0.39, 0.10)	0.244
Alcohol drinker	0.53 (0.31, 0.75)	**< 0.001**	0.02 (-0.19, 0.21)	0.915
BMI (kg/m^2^)				
Low Risk (Healthy)	1.00 (ref)		1.00 (ref)	
Underweight	0.22 (-0.28, 0.73)	0.380	0.09 (-0.33, 0.51)	0.676
Moderate Risk	-0.62 (-0.84, -0.41)	**< 0.001**	-0.37 (-0.55, -0.19)	**< 0.001**
High Risk	-1.24 (-1.46, -1.02)	**< 0.001**	-0.82 (-1.01, -0.62)	**< 0.001**
CHD	-1.04 (-1.35, 0.73)	**< 0.001**	-0.28 (-0.54, -0.03)	**0.034**
Hypercholesterolaemia	-1.33 (-1.50, -1.16)	**< 0.001**	-0.71 (-0.85, -0.56)	**< 0.001**
Tumours of any type	-0.31 (-0.05, 0.33)	0.350	-0.55 (-1.06, -0.04)	**0.036**
TIA/stroke	-1.48 (-2.31, -0.65)	**< 0.001**	-0.83 (-1.50, -0.16)	**0.015**

Bold values indicate p-values < 0.05

Model I: Univariate analysis

Model II: age, gender, education, ethnicity, education level, BMI, smoking, alcohol consumption, employment status, history of CHD/hypercholesterolaemia/ tumours of any type/ TIA/stroke

Table 3 presents the associations between HAI and four outcome measures: ABI, HGS, EQ-5D components, and K10 scores. In Model I, representing univariate analysis, all outcome measures displayed significant associations with HAI (p-values < 0.05). Adjusting for sociodemographic factors such as age, gender, ethnicity, and education level in Model II did not substantially modify the relationships between HAI and ABI, K10, HGS, and most EQ-5D components. However, the association between HAI and the self-care component of EQ-5D became non-significant. Notably, the association between HGS and HAI did not achieve statistical significance.

**Table 3:** Associations between outcomes measures and HAI

	Model I		Model II		Model III		Model IV	
	OR (95% CI)	*p*	OR (95% CI)	*p*	OR (95% CI)	*p*	OR (95% CI)	*p*
ABI	0.68 (0.59, 0.79)	**< 0.001**	0.74 (0.61, 0.88)	**0.001**	0.73 (0.61, 0.87)	**0.001**	0.73 (0.60, 0.88)	**0.001**
HGS (kg)	0.80 (0.76, 0.84)	**< 0.001**	0.96 (0.90, 1.02)	0.195	0.94 (0.89, 1.00)	0.070	0.95 (0.90, 1.02)	0.157
K10 score	0.88 (0.82, 0.94)	**< 0.001**	0.86 (0.80, 0.93)	**< 0.001**	0.87 (0.80, 0.94)	**0.001**	0.86 (0.79, 0.94)	**< 0.001**
EQ-5D Dimensions*								
Problems with mobility	0.74 (0.69, 0.79)	**< 0.001**	0.82 (0.76, 0.90)	**< 0.001**	0.85 (0.78, 0.93)	**< 0.001**	0.86 (0.79, 0.94)	**0.001**
Problems with self-care	0.83 (0.68, 1.00)	**0.044**	1.06 (0.84, 1.35)	0.621	1.05 (0.83, 1.36)	0.670	1.11 (0.86, 1.43)	0.417
Problems with usual activities	0.81 (0.72, 0.90)	**< 0.001**	0.87 (0.76, 1.00)	**0.039**	0.89 (0.77, 1.01)	0.080	0.89 (0.77, 1.02)	0.108
Having pain/ discomfort	0.89 (0.84, 0.93)	**< 0.001**	0.92 (0.86, 0.98)	**0.007**	0.93 (0.88, 1.00)	**0.039**	0.95 (0.89, 1.02)	0.168
Having depression/ anxiety	0.86 (0.81, 0.92)	**< 0.001**	0.87 (0.80, 0.94)	**0.001**	0.86 (0.79, 0.93)	**0.001**	0.88 (0.81, 0.96)	**0.003**

*Reference group: No problems

Bold values indicate p-values < 0.05

Model I: Univariate analysis

Model II: age, gender, ethnicity, education level

Model III: Model II + BMI + smoking + alcohol consumption + employment status

Model IV: Model III + history of CHD/hypercholesterolaemia/ tumours of any type/ TIA/stroke

Upon comprehensive covariate adjustment in Model IV, significant associations were sustained for ABI and K10 scores. Furthermore, the associations with the mobility and depression/anxiety components of EQ-5D retained their statistical significance. Nevertheless, while the associations with the usual activities and pain/discomfort components of EQ-5D persisted, they did not attain statistical significance.

## Discussion

In this multi-ethnic cohort, we found that older age, being unemployed, having moderate and high-risk levels of BMI, histories of CHD and hypercholesterolemia, and being of Malay or Indian ethnicity were associated with lower HAI scores, indicating a less healthy status. Conversely, being female and possessing higher education levels (secondary and tertiary) were associated with higher HAI scores, signifying better health. Furthermore, we found that the HAI was significantly associated with ABI, K10 scores and the mobility and depression/anxiety dimensions of EQ-5D.

The findings regarding the correlations of the HAI are consistent with existing literature. A study conducted in Singapore observed higher scores on an aging index associated with female sex and higher education levels [49]. Another Singaporean study exploring successful aging found weaker associations with older age and belonging to Malay or Indian ethnicities [50]. Additionally, studies from the UK [51] and China [52] reported similar associations between age, BMI, history CHD, sex, and education with higher scores on aging indices. Notably, sex and education consistently emerged as positive influences on the HAI, a trend that may be partly explained by the characteristics of developed countries. The factors examined in our study are significant determinants of health and represent known risk factors for various clinical diseases and health outcomes.

The components comprising the HAI are reflective of the primary health information from different physiological systems, making it align with other health markers [27, 28, 52]. This reinforces the association of factors such as age, BMI, lifestyle factors like smoking, and history of chronic conditions with the computation of HAI.

Beyond biological factors, socioeconomic status, including education and employment levels, play a substantial role in determining health outcomes, reflecting the social and lifestyle dimensions impacting overall health. Lower socioeconomic status is linked to heightened risks of poorer health outcomes and accelerated aging [53]. The association of Malay and Indian ethnicity with lower HAI scores could be attributed to the higher prevalence of co-morbidities like diabetes and hypertension among these groups compared to the Chinese population in Singapore [54]. This elevated prevalence might contribute to the lower HAI scores for Malay and Indian individuals compared to Chinese participants, given that the scoring component of HAI for the metabolic and cardiovascular systems includes self-reported histories of diabetes and hypertension diagnoses.

While previous research on HAI often explored its association with mortality, disability, mobility, gait speed, or incident cardiovascular diseases, there was a lack of data regarding the relationships between HAI and ABI, K10 scores, and HRQoL. The ABI serves as a diagnostic tool to effectively identify peripheral artery disease by measuring blood pressure in the ankle and arm [55]. Our study revealed a significant association between lower ABI values and lower HAI scores, indicating an overall less healthy state. This relationship can be understood through the pathophysiology of peripheral artery disease, which involves the accumulation of atherosclerotic plaque along arterial walls [56]. As the plaque thickens, it restricts arterial dilation, leading to impaired blood flow, arterial narrowing, and reduced blood pressure in the ankle arteries. Previous studies have also demonstrated significant negative correlations between brachial systolic blood pressure and ABI [57, 58]. This underscores the utility of ABI as part of HAI for predicting PAD diagnoses especially in younger adults who are at risk of premature PAD. Premature PAD is characterised by disease diagnosis before 50 years of age and has seen a rise globally and is often undiagnosed [59].

The relationship between EQ-5D dimensions and HAI displayed a significant negative correlation, particularly evident in the mobility and depression/anxiety components. This finding aligns with the observed results for the K10 scores. Moreover, the distribution of EQ-5D index scores demonstrated lower average scores among individuals with lower HAI scores. The association between mobility and HAI has been explored in previous studies, revealing that unhealthier HAI scores are linked to slower gait speed [19]. A study conducted in China, utilizing a modified version of HAI, demonstrated significant associations between HAI scores and both objective and self-reported mobility parameters [30]. These findings underscore the utility of HAI as an evaluation method for early intervention in addressing mobility limitations. This alignment between HAI, EQ-5D, and mobility further emphasizes the comprehensive nature of the HAI in reflecting an individual’s health status across various dimensions. The significant associations observed between HAI and these outcome measures highlight the potential of HAI as a valuable evaluation method for assessing and predicting health-related outcomes and identifying areas of intervention.

As functional mobility declines, a wealth of studies have consistently demonstrated that quality of life tends to decrease. The presence of co-morbidities or diseases can result in both functional and cognitive decline, leading to an increased reliance on activities of daily living. Research with older adults suffering from multiple chronic conditions has revealed that several factors negatively impact HRQoL, including a high symptom burden, diminished ability to perform ADLs, and a higher prevalence of depression [60]. Furthermore, studies have indicated that older adults with better mobility tend to experience a higher quality of life, whereas those with health issues that limit mobility experience a significant decline in HRQoL [61]. These observations may be attributed to alterations in lifestyle and a lack of social support in terms of well-being and societal interactions, both of which contribute to a lower HRQoL [62].

HRQoL encompasses both physical and mental health dimensions, and factors that impact HRQoL are likely to influence depression symptoms, which are measured by the K10 score. Therefore, it is reasonable to anticipate that these factors would play a primary role in individuals scoring poorly on the HAI, subsequently leading to reported challenges in the EQ-5D-5 L and elevated K10 scores. The notable influence on the mobility and depression/anxiety dimensions of the EQ-5D might be particularly pronounced within the Singaporean population, offering an explanation for the significant associations observed in this study.

In our study, the absence of a significant association between HAI and the self-care, usual activities, and pain/discomfort dimensions of EQ-5D may be attributed to the intricate interplay of diverse clinical, physical, and social factors that collectively influence HRQoL. HRQoL acts as a comprehensive reflection of an individual’s overall health, and its complex nature could have contributed to the lack of significant associations in these particular aspects of the EQ-5D. Furthermore, we noted that the self-care component of EQ-5D was attenuated in the final model. This attenuation might be explained by the advanced age of our study population, as a substantial portion of participants were over the age of 60 and could potentially require assistance with daily tasks like bathing and dressing. Additionally, non-health-related factors such as financial or social issues could have influenced the self-care component of EQ-5D.

Handgrip strength (HGS) serves as a widely utilized marker for overall muscle strength. Reduced handgrip strength (HGS) is commonly linked to the physiological aging process [61] and is associated with compromised functional abilities and an increased risk of dependency [63, 64]. HGS serves as a significant biomarker of health, offering insights into an individual’s overall health status [65–67]. Numerous studies have highlighted the association of HGS with various health conditions such as cardiovascular disease [68, 69], diabetes [70, 71], and psychological issues [72]. In our study, we observed direct relationship between HGS and Healthy Ageing Index (HAI) scores, consistent with previous research in Singapore indicating that HGS decreases with advancing age [73]. This relationship aligns with HGS recognized utility as a biomarker of health, whereby lower HAI scores, reflecting poorer health outcomes, were associated with decreasing HGS values. However, it is worth noting that this association did not reach statistical significance in our analysis. The absence of statistical significance could potentially be influenced by a range of factors, including the age distribution within our study population (which encompassed individuals aged 40 years and older) and potential variations in HGS measurements due to methods that might not be user-specific. The age range of our study participants might have contributed to the lack of significance in the association. Muscle strength, as measured by HGS, can be influenced by age-related changes in muscle mass and overall physiological function. Given the inclusion of participants spanning from 40 years and older, there could be variations in HGS based on the age distribution, potentially impacting the statistical significance of the relationship.

The strength of our study include: the first population-based study in Singapore to investigate the association between HAI and four important health-related outcomes: peripheral arterial disease, overall muscle strength, HRQoL, and psychological distress. Furthermore, our study took into account various potential confounding factors by performing adjustments during the regression analysis. This ensures that the observed associations between HAI and the four outcomes are more robust and reliable.

The study also has its limitations. First, our study population primarily consisted of individuals who were relatively healthy at baseline. This may potentially restrict the generalizability of our findings to populations with a higher prevalence of health conditions or comorbidities. The outcomes and HAI components being based on binary outcomes could pose a limitation as well, especially given that a majority of participants fell within the normal range for all four outcomes. This could result in reduced sensitivity and statistical power, possibly affecting our ability to identify significant differences or associations. Second, the reliance on self-reported data for some covariates used in the models and for the computation of HAI introduces the potential for recall bias or misclassification. This introduces the possibility of underreporting by participants, resulting in information which may potentially mask the true effects of the associations examined. Furthermore, the selection of cut-off points for both the outcomes and HAI computation is inherently subjective. While these cut-off points were chosen based on a comprehensive literature review, their standardization or validation for application in the broader population of Singapore may vary. Moreover, while we prioritized the use of validated instruments, the K10 questionnaire has not been validated in Singapore. This might impede the generalizability of our findings and introduce an element of uncertainty. Our study employed a complete case analysis where only participants with complete data for all variables of interest were included. Whilst this approach ensures data quality, it is crucial to acknowledge a potential limitation of this approach. Given that the two outcomes of interest i.e. EQ-5D and MMSE were missing in a substantial number of individuals, excluding individuals with this data may introduce reverse causality. It is essential to interpret the results cautiously, considering the impact of missing data on the generalizability and reliability of our findings. Finally, there may be residual confounding due to unknown factors (such as other comorbidities) that are more prevalent in the Malay and Indian ethnicities, contributing to the observed association.

## Conclusion

In summary, our study found that lower HAI scores which are indicative of unhealthier states, are associated with factors such as older age, Malay and Indian (compared with Chinese) ethnicity, unemployed, moderate and high-risk levels of BMI and heart disease, hypercholesterolemia, tumours of any type and stroke history. Higher HAI scores was associated with female sex and higher education levels. Future research could explore how the HAI evolves over time in relation to different health outcomes and across diverse ethnic groups. Longitudinal studies tracking individuals over extended periods could help uncover trends and patterns in the development of age-related diseases and disorders, as well as changes in overall health status. Such investigations would contribute substantially to advancing our comprehension of healthy aging and informing effective healthcare approaches.

### Electronic supplementary material

Below is the link to the electronic supplementary material.

Supplementary Material 1

## Data Availability

The datasets used and/or analysed during the current study available from the corresponding author on reasonable request.
