# Supplementary material for: Association of a healthy ageing index with health-related outcomes in a multi-ethnic cohort from Singapore
Source: BMC Geriatr. 2024 Jun 11;24:508. doi: 10.1186/s12877-024-05099-7 (PMC11165847; doi:10.1186/s12877-024-05099-7)
Supplement: Supplementary file 1 — Supplementary Material 1 [file 12877_2024_5099_MOESM1_ESM.docx]

## **SUPPLEMENTARY TABLES**

## **Supplementary table 1: Cut-off points used for Healthy Ageing Index components**

| Healthy Ageing Index: (unhealthiest) 0 to 10 (most healthy) | | |
| --- | --- | --- |
| Component | Marker | Values |
| Cardiovascular | Systolic BP (mmHg) | 2: <126  1: 126 -143  0: ≥143 or Diagnosis (Hypertension) or Medication history |
| Respiratory | Self-reported a diagnosis of chronic lung disease (including pulmonary tuberculosis, chronic bronchitis, asthma, or COPD) | 2: No pulmonary disease  0: Any diagnosis of pulmonary disease |
| Metabolic | Fasting blood glucose (mmol/L) | 2: ≤5.5  1: 5.6-7.0  0: ≥7.0 or Diagnosis (DM) or Medication history |
| Urinary | Creatinine (mmol/L) | 2: <97.2 (M), <70.7 (F)  1: 97.2-114.9 (M), 70.7-88.4 (F)  0: ≥114.9 (M), ≥88.4 (F) |
| Neurological | MMSE | 2: ≥26 (cognitively normal)  0: ≤25 (Having cognitive impairment) |

## **Supplementary table 2:** Cut-off points used for binary variables of outcomes

| Outcomes | Marker Tests | Binary Values  (0: Normal, 1: Abnormal) |
| --- | --- | --- |
| Peripheral Artery Disease (PAD) | Ankle-Brachial Index | 0: ABI>0.9 (Normal)  1: ABI ≤ 0.9 |
| Overall muscle strength | Handgrip strength | 0: >20kg for females, >30kg for males  1: ≤20kg for females, ≤30kg for males |
| Health-related Quality of Life (HRQoL) | EQ-5D-5L questionnaire that measures HRQoL on 5 dimensions:  (i) mobility  (ii) self-care  (iii) usual activities  (iv) pain/discomfort  (v) anxiety/depression | 0: No problems  1: At least some problems |
| Psychological distress | Kessler Psychological Distress scale (K10) | 0: K10 Score ≤19 (Well)  1: K10 Score ≥ 20 |

**Supplementary Table 3: Baseline characteristics of included participants by ethnicity (N=1909)**

|  | Chinese | Malay | Indian | p-Value* |
| --- | --- | --- | --- | --- |
|  | N = 757 | N = 478 | N = 674 |  |
| Age (min age =40) | 52 (47, 59) | 55 (49, 60) | 55 (48, 63) | <0.001 |
| Female | 443 (58.5) | 295 (61.7) | 392 (58.2) | 0.428 |
| Education |  |  |  | <0.001 |
| Primary | 158 (20.9) | 162 (33.9) | 227 (33.7) |  |
| Secondary | 291 (38.4) | 224 (46.9) | 266 (39.5) |  |
| Tertiary | 308 (40.7) | 92 (19.2) | 181 (26.8) |  |
| Full time/part-time employment | 579 (76.5) | 275 (57.5) | 436 (64.7) | <0.001 |
| Smoking | 145 (19.2) | 116 (24.3) | 125 (18.6) | 0.038 |
| Alcohol consumption | 236 (31.2) | 2 (0.42) | 124 (18.4) | <0.001 |
| BMI (kg/m^2^) | 23.3 (21.2, 26.0) | 27.5 (24.4, 31.3) | 26.4 (23.6, 29.7) | <0.001 |
| History of CHD^a^ | 42 (5.6) | 33 (6.9) | 87 (12.9) | <0.001 |
| History of hypercholesterolaemia | 258 (34.1) | 226 (47.3) | 330 (49.0) | <0.001 |
| History of tumour of any type | 17 (2.3) | 9 (1.9) | 10 (1.5) | 0.571 |
| History of TIA/stroke | 8 (1.1) | 6 (1.3) | 7 (1.0) | 0.931 |
| HAI score | 9 (8,10) | 8 (6, 9) | 8 (6, 9) | <0.001 |
| ABI | 1.08 (1.02, 1.13) | 1.01 (1.06, 1.12) | 1.05 (1.00, 1.11) | <0.001 |
| HGS (kg) | 23.5 (18.6, 30.2) | 20.8 (16.6, 28.1) | 16.7 (21.8, 28.7) | <0.001 |
| K10 score | 12 (10, 15) | 12 (10, 15) | 13 (11, 18) | <0.001 |
| MMSE score | 29 (28, 30) | 29 (27, 30) | 29 (26, 30) | <0.001 |

Data are presented as median (Q1, Q3) for continuous variables unless stated otherwise as n (%) for categorical variables.

**p*-value was obtained via chi-square for categorical variables and Kruskal-Wallis test for continuous variables with a *p*-value of <0.05 taken to be statistically significant.

ABI: Ankle-brachial index, BMI: Body Mass Index, CHD: Coronary Heart Disease, HAI: Healthy Ageing Index, HGS: Handgrip strength, K10: Kessler10 Psychological Distress Scale, MMSE: Mini-Mental State Examination, TIA: Trans-ischemic attack

^a^ CHD: Heart failure, heart attack, history of angioplasty, insertion of balloons and stents and valve prolapse
